# CRISPR/Cas9 Gene Editing of *NtAITRs*, a Family of Transcription Repressor Genes, Leads to Enhanced Drought Tolerance in Tobacco

**DOI:** 10.3390/ijms232315268

**Published:** 2022-12-03

**Authors:** Guimin Li, Yanxing Ma, Xiaoping Wang, Nini Cheng, Deyu Meng, Siyu Chen, Wei Wang, Xutong Wang, Xiaojun Hu, Li Yan, Shucai Wang

**Affiliations:** 1 Laboratory of Plant Molecular Biology & Crop Gene Editing, School of Life Sciences, Linyi University, Linyi 276000, China; 2 Key Laboratory of Molecular Epigenetics of MOE, Northeast Normal University, Changchun 130024, China

**Keywords:** NtAITRs, transcription factors, tobacco, ABA, drought stress, abiotic stress, gene editing

## Abstract

Tobacco is a cash crop throughout the world, and its growth and development are affected by abiotic stresses including drought stress; therefore, drought-tolerant breeding may help to improve tobacco yield and quality under drought stress conditions. Considering that the plant hormone ABA (abscisic acid) is able to regulate plant responses to abiotic stresses via activating ABA response genes, the characterization of ABA response genes may enable the identification of genes that can be used for molecular breeding to improve drought tolerance in tobacco. We report here the identification of NtAITRs (*Nicotiana tabacum* ABA-induced transcription repressors) as a family of novel regulators of drought tolerance in tobacco. Bioinformatics analysis shows that there are a total of eight *NtAITR* genes in tobacco, and all the NtAITRs have a partially conserved LxLxL motif at their C-terminus. RT-PCR results show that the expression levels of at least some *NtAITRs* were increased in response to ABA and drought treatments, and NtAITRs, when recruited to the *Gal4* promoter via a fused GD (*Gal4* DNA-binding domain), were able to repress transcription activator LD-VP activated expression of the *LexA-Gal4-GUS* reporter gene. Roles of NtAITRs in regulating drought tolerance in tobacco were analyzed by generating CRISPR/Cas9 gene-edited mutants. A total of three Cas9-free *ntaitr12356* quintuple mutants were obtained, and drought treatment assays show that drought tolerance was increased in the *ntaitr12356* quintuple mutants. On the other hand, results of seed germination and seedling greening assays show that ABA sensitivity was increased in the *ntaitr12356* quintuple mutants, and the expression levels of some ABA signaling key regulator genes were altered in the *ntaitr12356-c3* mutant. Taken together, our results suggest that *NtAITRs* are ABA-responsive genes, and that NtAITRs function as transcription repressors and negatively regulate drought tolerance in tobacco, possibly by affecting plant ABA response via affecting the expression of ABA signaling key regulator genes.

## 1. Introduction

Environmental stresses including abiotic stresses and biotic stresses reduce the yield of plants, including crops [1,2]. It is estimated that biotic stresses such as pathogens and insects cause ~20% whereas abiotic stresses such as drought and salt cause ~50% of global yield loss for most major crops [3,4]. On the other hand, to survive the abiotic stress conditions they are constantly exposed to, plants have evolved mechanisms in response to abiotic stresses [5], and dissection of the mechanisms underlining plant responses to abiotic stresses may lead to the breeding of crops with enhanced abiotic stress tolerance, therefore increasing crop yield under abiotic stress conditions. 

It is proposed that plants respond to abiotic stresses via two different signaling pathways, i.e., the plant hormone ABA (abscisic acid)-dependent and -independent pathways [5]. As a key stress hormone, ABA accumulates dramatically in response to abiotic stresses such as drought and salt, leading to the activation/repression of ABA response genes via signaling transduction through several key regulators, including the PYR1/PYL/RCAR (Pyrabactin resistance 1/PYR1-like/regulatory component of ABA receptor) ABA receptors, the ABA signaling negative regulators A-group PP2C (PROTEIN PHOSPHATASE 2C) phosphatases, the ABA signaling positive regulators SnRK2 (NONFERMENTING 1 (SNF1)-RELATED PROTEIN KINASES) protein kinases, and downstream ABF/AREB/ABI5 type bZIP (basic region leucine zipper) transcription factors, thereby affecting plant responses to abiotic stresses [6,7,8,9,10,11,12,13]. 

Consistent with this, changes in expression levels of the ABA signaling key regulator genes including the *PYR1/PYL/RCAR* receptor genes, the *PP2C* phosphatase genes, the *SnRK2* protein kinase genes and the *ABF/AREB/ABI5* transcription factor genes affected plant responses to ABA, resulting in changes in abiotic stress tolerance in plants [14,15,16,17]. Similarly, changes in expression levels of protein stability regulator genes of some ABA signaling key regulators, including the E2 ligase gene *VPS23A* and E3 ligase genes *CUL4*, *RSL1*, *KEG* (*KEEP ON GOING*), *DWA1*, *DWA2*, *PUB12* and *PUB13*, are also able to affect plant response to ABA and thereby plant responses to abiotic stresses [18,19,20,21,22,23,24,25,26]. In addition, changes in expression levels of ABA response genes such as the MYB transcription factor genes *MYB44* and *MYB71*, the bHLH transcription factor gene bHLH112, the WD40 protein genes *AIW1* (*ABA Induced WD40-repeat 1*) and *AIW2*, and heat shock factor gene *HSFA6b* also affected plant responses to abiotic stresses [27,28,29,30,31]. Considering that there are hundreds of thousands of ABA-responsive genes remained uncharacterized, it is very likely that some of the genes may be involved in the regulation of plant responses to abiotic stresses. 

In an attempt to identify novel regulators of plant abiotic stress responses from unknown-function ABA response genes, we identified AITRs (ABA-induced transcription repressors), a novel family of transcription repressors in Arabidopsis as negative regulators of plant abiotic stress responses [12,13]. We found that the Arabidopsis *aitr* mutants showed enhanced tolerance to abiotic stresses including drought and salt [12], and knockout of the entire family of *AITRs* did not affect plant growth and development in Arabidopsis [13]. Considering that AITRs are conserved in angiosperms [12], *AITRs* may be good candidate genes for molecular breeding to improve abiotic stress tolerance in crops. Indeed, we found that AITRs in several different crops including tomato, rice, soybean and cotton shared similar features with Arabidopsis AITRs, i.e., their gene expression is induced by ABA, and they function as transcription repressors [12,32,33]. In addition, expression of cotton *GhAITR-A1* restored the enhanced abiotic stress tolerance in *aitr1* mutants [32], indicating that GhAITRs may also function as negative regulators of plant abiotic stress tolerance. Most importantly, knockout of several *GmAITR* genes by CRISPR/Cas9 gene editing moderately enhanced salt tolerance in soybean [33]. However, we also note that *gmaitr* mutants showed slightly enhanced ABA sensitivity, a phenotype opposite to that of the Arabidopsis *aitr* mutants, which showed greatly decreased ABA sensitivity [12,33]. These results suggested that AITRs in different plants may have different functions at least in some aspects.

Tobacco (*Nicotiana tabacum*) is an economically important crop throughout the world, which is usually grown for smoking, but its biomass can also be a good source of advanced biofuels [34,35]. Similar to other plants, tobacco’s growth and development is also affected by environment stresses including abiotic stresses [36,37,38,39,40,41], and some studies have found that under abiotic stress conditions, numerous physiological and molecular changes in tobacco including accumulation of ABA were observed [39,40,41]. Studying the mechanisms of stress tolerances and mining regulatory genes involved in regulating abiotic stress responses, including the ABA response genes in tobacco, are essential for molecular breeding to improve abiotic stress tolerance in tobacco.

In this study, we provide evidence that tobacco AITRs (NtAITRs) are involved in the regulation of plant drought tolerance in tobacco. We found that the expression levels of at least some of the *NtAITRs* increased in response to ABA and drought treatment, and NtAITRs function as transcription repressors. By generating *ntaitr* mutants using CRISPR/Cas9 gene editing and examining their responses to ABA and abiotic stresses, we found that NtAITRs negatively regulate drought tolerance in tobacco via affecting the plant responses to ABA; therefore, *NtAITRs* may be targeted for CRISPR/Cas9 gene editing to improve drought tolerance in tobacco.

## 2. Results

### 2.1. Identification of AITRs in Tobacco

By using full-length amino acid sequences of Arabidopsis AITRs for *N. tabacum* Protein BLASTing on NCBI (https://blast.ncbi.nlm.nih.gov/Blast.cgi, accessed on 30 October 2018), we identified a total of eight AITRs in tobacco, i.e., NtAITR1 (LOC107760464), NtAITR2 (LOC107761277), NtAITR3 (LOC107767518), NtAITR4 (LOC107770126), NtAITR5 (LOC107791551), NtAITR6 (LOC107804873), NtAITR7 (LOC107815750) and NtAITR8 (LOC107829869). The coding sequences of the *NtAITRs* were obtained by using the locus numbers for gene searching on the KEGG GENES Database (https://www.genome.jp/kegg/genes.html, accessed on 30 October 2018), and corresponding genome sequences were then obtained by BLASTing the *N. tabacum* genome with the coding sequences on the Sol Genomics Network (https://solgenomics.net/organism/Nicotiana_tabacum/genome, accessed on 30 October 2018). We found that two of the *NtAITRs*, i.e., *NtAITR2* and *NtAITR7*, have two exons, and the other six *NtAITRs* have a single exon (Figure S1), whereas all the AITRs in Arabidopsis and soybean have only one exon [12,33]. 

**Figure 1:**
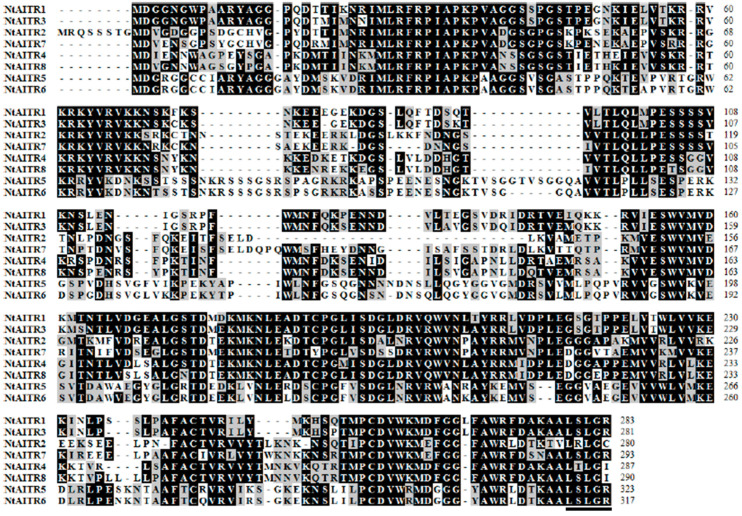
Amino acid sequence alignment of NtAITRs. Full-length amino acid sequences of the NtAITRs were obtained on NCBI (https://blast.ncbi.nlm.nih.gov/Blast.cgi, accessed on 30 October 2018) and used for multiple sequence alignment by using BioEdit. Identical amino acids are shaded in black and similar amino acids are shaded in gray. Underline indicates the partially conserved LxLxL transcriptional repression motif.

Amino acid sequence alignment shows that NtAITRs shared high identity and similarity, especially between each pair of the proteins, i.e., NtAITR1 and NtAITR3, NtAITR2 and NtAITR7, NtAITR4 and NtAITR8, and NtAITR5 and NtAITR6, but all of them have only a partially conserved LxLxL motif at the C-terminus (Figure 1). 

**Figure 2:**
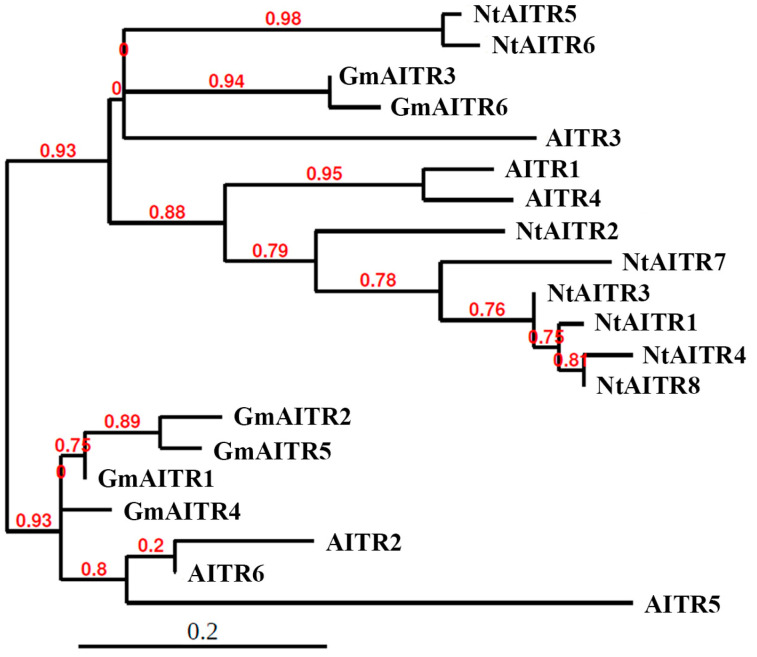
Phylogenetic analysis of NtAITRs, GmAITRs and Arabidopsis AITRs. Full-length amino acid sequences of GmAITRs and Arabidopsis AITRs were obtained from Phytozome (https://phytozome-next.jgi.doe.gov, accessed on 30 October 2018) and subjected to phylogenetic analysis with NtAITRs on phylogeny.fr (http://www.phylogeny.fr, accessed on 1 September 2022) by using the “One Click” mode with default settings. Branch support values are indicated above the branches.

Protein domain assays show that there are five conserved domains in all the NtAITRs, including one with the partially conserved LxLxL motif (Figure S2). Phylogenic analysis of AITRs from tobacco, Arabidopsis and soybean shows that NtAITRs formed one clade with three Arabidopsis AITRs (AITR1, AITR3 and AITR4) and two soybean AITRs (GmAITR3 and GmAITR6), whereas the other three Arabidopsis AITRs (AITR2, AITR5 and AITR6) and the other four soybean AITRs (GmAITR1, GmAITR2, GmAITR4 and GmAITR5) formed another clade (Figure 2). In addition, the phylogenic analysis also shows that NtAITR5 and NtAITR6 are closely related, and the other six NtAITRs are closely related (Figure 2).

### 2.2. Expression Levels of NtAITRs Are Increased in Response to ABA and Drought Treatments 

**Figure 3:**
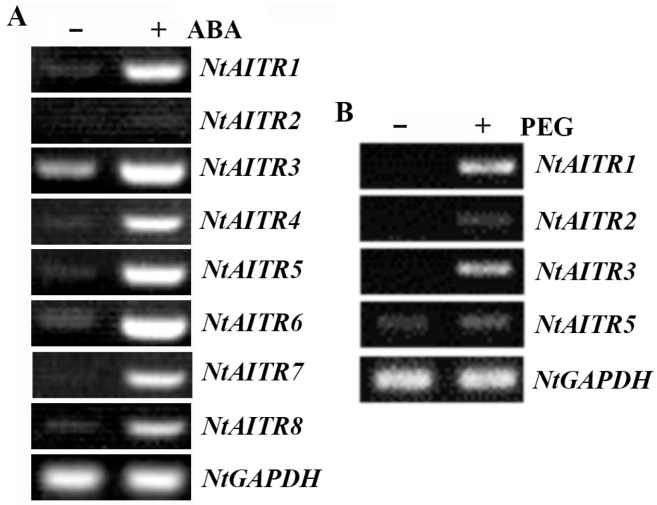
Expression of *NtAITRs* in response to ABA and drought treatments. (**A**) Expression of *NtAITRs* in response to ABA treatment. Ten-day-old K326 wild type tobacco seedlings were treated with 50 µM ABA for 4 h, then RNA was isolated and used for RT-PCR analysis to examine the expression of *NtAITRs*. The expression of *NtGAPDH* gene was used as a control. (**B**) Expression of *NtAITRs* in response to drought treatment. Twenty-day-old soil-grown K326 wild type seedlings were watered with 20% PEG6000, seedlings were collected 6 h after and then RNA was isolated and used for RT-PCR analysis to examine the expression of *NtAITRs*. The expression of *NtGAPDH* gene was used as a control.

Thus far, it has been shown that all the *AITRs* characterized from different plants including the model plant Arabidopsis, and crops including tomato, rice, soybean and cotton, are all inducible by ABA treatment, and all the AITRs function as transcription repressors [12,32,33]. To examine if that is also the case with *NtAITRs*, we first examined their expression in response to ABA treatment. Tobacco seedlings if the K326 wild type were treated with ABA for a short time, and the expression of NtAITRs was examined by RT-PCR. As shown in Figure 3A, it is clear that the expression levels of all the *NtAITRs* but *NtAITR2* were increased in the ABA-treated samples, as a very faint band was obtained for *NtAITR2* in the ABA-treated sample, which indicates that it may respond slightly to ABA treatment. We also examined the expression of *NtAITRs* in response to drought treatment by using PEG treatment to mimic drought condition, and we found that the expression levels of at least some *NtAITRs* including *NtAITR1*, *NtAITR2*, *NtAITR3* and *NtAITR5* were increased in response to drought treatment (Figure 3B).

**Figure 4:**
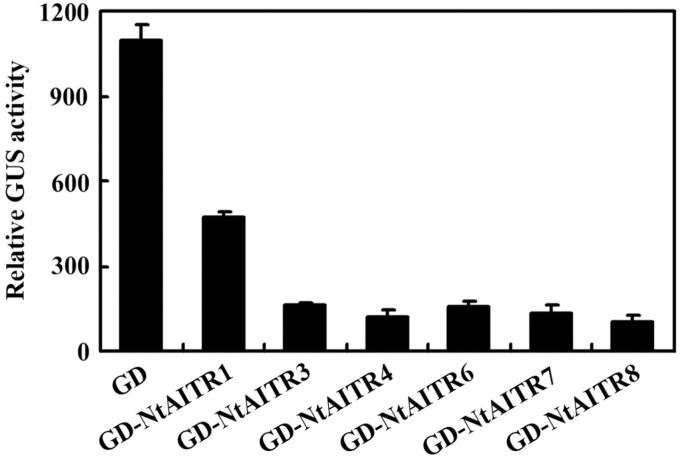
Transcription activities of NtAITRs. Plasmids of the *LexA-Gal4:GUS* reporter and the *LD-VP* activator were co-transfected with the *GD-NtAITR* effectors into protoplasts isolated from leaves of the Col wild type Arabidopsis. The transfected protoplasts were incubated in darkness for 20–22 h, and then GUS activity was assayed. Co-transfection of *GD* was used as a control. Data represent the mean ± SD of three replicates.

We then examined the transcription activities of NtAITRs by using protoplast transfection assays. We successfully cloned full-length CDS for *NtAITR1*, *NtAITR3*, *NtAITR4*, *NtAITR6*, *NtAITR7* and *NtAITR8*, and generated constructs with a fused N-terminal GD tag. Plasmids of the *GD-NtAITR* constructs were co-transfected with plasmids of the transcription activator gene *LD-VP* and the *LexA-Gal4:GUS* reporter gene into protoplasts isolated from Col wild type Arabidopsis leaves. As shown in Figure 4, LD-VP-activated GUS expression was repressed when *GD-AITRs* were co-transfected, suggesting that all the NtAITRs examined function as transcription repressors. 

### 2.3. Generation of Transgene-Free Ntaitr Mutants

The above results show that NtAITRs shared similar features with AITRs characterized from other plants including Arabidopsis, soybean, tomato, cotton and rice, i.e., their gene expression is induced by ABA, and they function as transcription repressors. These results indicate that NtAITRs may also be involved in the regulation of ABA and abiotic stress responses in tobacco. To examine if this is indeed the case, we decided to generate *ntaitr* mutants by using CRISPR/Cas9 gene editing. 

**Figure 5:**
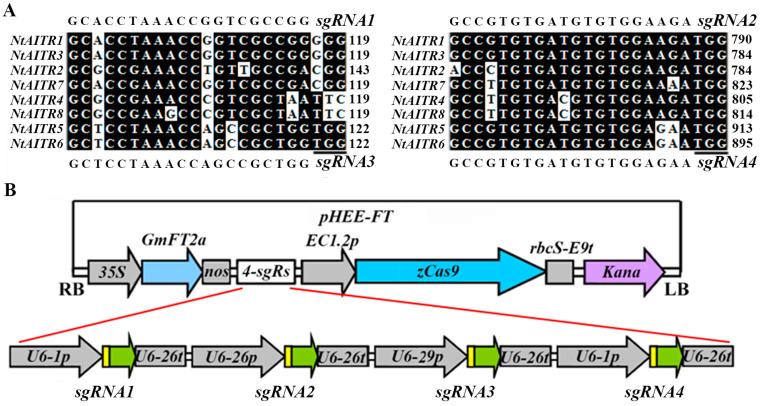
Generation of an *FT* expression cassette containing *pHEE* CRISPR/Cas9 construct for *NtAITRs* editing. (**A**) Target sequences inserted into the sgRNAs and alignment of the sequences may be targeted in *NtAITRs* by using Bioedit. Numbers indicate the nucleotide position relative to the first nucleotide in the coding sequence of *NtAITRs*, underlines indicate PAM sites. (**B**) Diagram of the *pHEE* vector with an *FT* expression cassette and four sgRNAs. Target sequences that can target *NtAITR1*, *NtAITR2*, *NtAITR3*, *NtAITR5* and *NtAITR6* were introduced into the sgRNA expression cassettes by PCR amplification, followed by Golden Gate reaction with the *pHEE-FT* vector.

Considering that *NtAITRs* shared high nucleotide sequence identity, especially between each pair of the genes, i.e., *NtAITR1* and *NtAITR3*, *NtAITR2* and *NtAITR7*, *NtAITR4* and *NtAITR8*, and *NtAITR5* and *NtAITR6* (Figure S3), we selected four different target sequences that may simultaneously target several different *NtAITRs*, including *NtAITR1*, *NtAITR2*, *NtAITR3*, *NtAITR5* and *NtAITR6* (Figure 5A), and made an *FT* expression cassette containing the CRISPR/Cas9 construct with four sgRNAs (Figure 5B). The containing of the *FT* expression cassette enables the reduction in the time required to generate gene-edited mutants and facilitates the selection of Cas9-free mutants [42]. By using the construct to transform wild-type tobacco, we successfully obtained some transgenic plants with early flowering phenotypes. Gene editing status in these early flowering plants was examined, and transgene-free mutants were obtained in next generation based on flowering phenotype, then confirmed by PCR amplification of the *Cas9* fragment, and gene editing status was examined again. 

**Figure 6:**
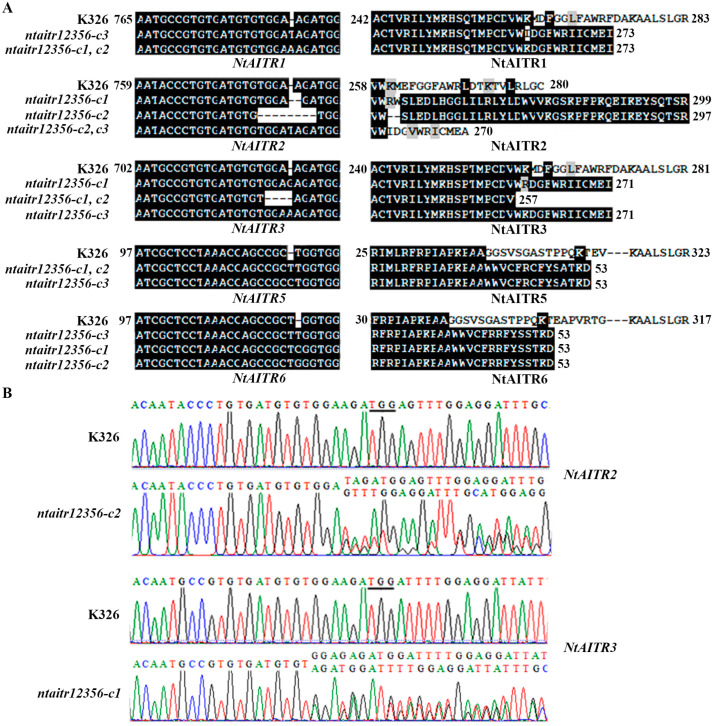
Generation of the *ntaitr12356* quintuple mutants. (**A**) Alignment of DNA sequences (**left**) and amino acid sequences (**right**) at the target sites of NtAITR1, NtAITR2, NtAITR3, NtAITR5 and NtAITR6, respectively, in the K326 wild type and the *ntaitr12356* quintuple mutants. The CRISPR/Cas9 construct was transformed into the K326 wild type tobacco, gene editing status was examined in the early flowering T0 plants and transgene-free homozygous mutants were isolated in T1 or T2 generation of the edited T0 plants. Numbers indicate the nucleotide position relative to the first nucleotide of *NtAITRs*, or the first amino acid of NtAITRs. (**B**) Editing status of *NtAITR2* in the *ntaitr12356-c2* mutant and *NtAITR3* in the *ntaitr12356-c1* mutant. Underlines indicate PAM sites.

Three transgene-free homozygous quintuple mutants, i.e., *ntaitr1 ntaitr2 ntaitr3 ntaitr5 ntaitr6-c1* (*ntaitr12356-c1*), *ntaitr12356-c2* and *ntaitr12356-c3* were obtained. In all the mutants, either a single-nucleotide insertion/deletion or the deletion of a few nucleotides occurred at the target sites of the *NtAITRs*, resulting in a frame shift after the edited sites of the corresponding NtAITRs (Figure 6A). It should be noted that *NtAITR2* in *ntaitr12356-c2* and *NtAITR3* in *ntaitr12356-c2* were edited in a bioallelic manner (Figure 6B), but either way resulted in a frame shift (Figure 6A); therefore, seeds collected from the mutants were directly used for the following experiments.

### 2.4. The ntaitr12356 Mutant Plants Show Enhanced Tolerance to Drought

**Figure 7:**
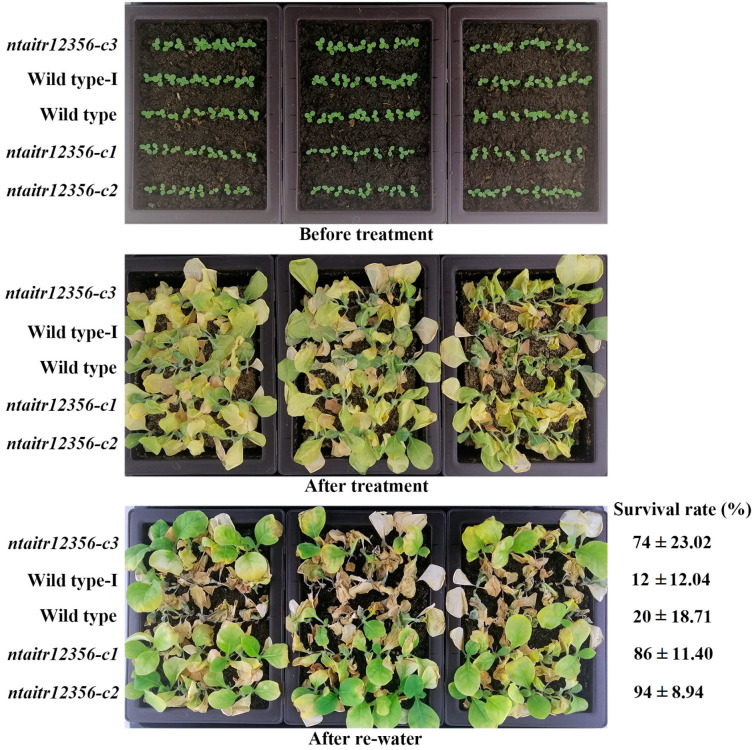
Drought tolerance of the *ntaitr12356* quintuple mutants. Seeds of the K326 wild type, the wild type-I and the *ntaitr12356* mutants were germinated in soil trays and grown for 10 days before drought treatment. Drought treatment was performed by withholding watering for 20 days, and then watering was resumed. Pictures were taken before drought treatment, after drought treatment and 5 days after watering was resumed. Plants that survived were counted 5 days after watering was resumed, and survival rate was calculated. Data represent the mean ± SD of five replicates.

Expression level changes of *AITRs* in both Arabidopsis and soybean affected plant response to abiotic stresses [12,13,33]. After showing that NtAITRs shared similar features with AITRs from other plants examined so far, we examined drought tolerance in the *ntaitr12356* quintuple mutants. The K326 wild type tobacco and the wild type-I were used as controls. The wild type-I is a non-edited plant segregated from the offspring of the T0 plant from which the *ntaitr12356-c3* quintuple mutant was isolated. Seeds of the wild type, the wild type-I and the *ntaitr12356* quintuple mutants were germinated in soil pots and grown in a growth room. Ten days after germination, the plants were watered thoroughly, and then watering was withheld for drought treatment. Twenty days after withholding watering, watering was resumed. As shown in Figure 7, the seedlings of the wild type, the wild type-I and the *ntaitr12356* quintuple mutants were largely similar before the drought treatment. All the plants continued growing during the treatment, but most of the leaves of the plants nearly dried after the drought treatment, with the three *ntaitr12356* quintuple mutants to a less severe degree (Figure 7). Five days after watering was resumed, at least four-fifths of each of the three *ntaitr12356* quintuple mutants survived, whereas at least four-fifths of the wild type and the wild type-I plants died, and no big difference was observed among the three *ntaitr* quadruple mutants or between the wild type and the wild type-I plants (Figure 7), indicating that drought tolerance was enhanced in the *ntaitr12356* quintuple mutants.

### 2.5. The ntatir12356 Mutants Are Hypersensitive to ABA

**Figure 8:**
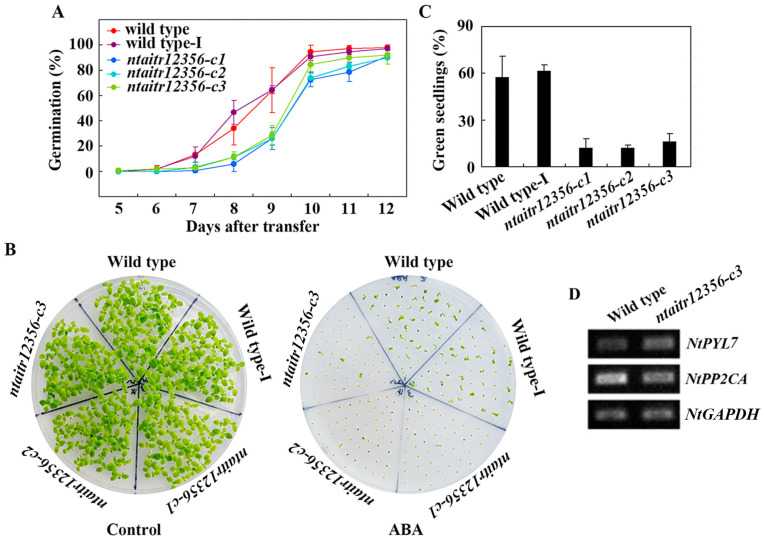
ABA sensitivity of the *ntaitr12356* quintuple mutants. (**A**) Seed germination of the K326 wild type, the wild type-I and the *ntaitr12356* quintuple mutants in response to ABA. Sterilized seeds the K326 wild type, the wild type-I and the *ntaitr12356* mutants were kept at 4 °C in darkness for 7 days and then sown on 1/2 MS plates in the presence or absence of 2 μM ABA. The plates were transferred to a growth room and seeds germinated were counted daily after the plates were transferred. Data represent the mean ± SD of three replicates. (**B**) Seedling greening of the K326 wild type, the wild type-I and the *ntaitr12356* quintuple mutants in response to ABA. Pictures were taken 12 days after the plates were transferred. (**C**) Percentage of green seedlings of the K326 wild type, wild type-I and the *ntaitr12356* quintuple mutants in response to ABA. Green seedlings were counted 12 days after the transfer, and the percentage of green seedlings was calculated. Data represent means ± SD of three replicates. (**D**) Expression of *NtPYL7* and *NtPP2CA* in the K326 wild type and the *ntaitr12356-c3* quintuple mutant seedlings in response to ABA. Ten-day-old seedlings were treated with 50 µM ABA or solvent methanol as a control for 4 h. Total RNA was isolated, and RT-PCR was used to examine the expression of the ABA signaling component genes. The expression of *NtGAPDH* was used as a control.

After showing that drought tolerance was enhanced in *ntaitr12356* quintuple mutants, we further examined if this may be caused by alternated ABA response in the mutants. Seed germination and cotyledon greening assays were used to examine ABA sensitivity of the *ntaitr12356* quintuple mutants, and the K326 wild type tobacco and the wild type-I were used as controls. As shown in Figure 8A, in the seed germination assays, all three *ntaitr12356* quintuple mutants showed a clearly increased sensitivity to ABA compared to the wild type and the wild type-I. We also note that little if any difference on the germination rate was observed among the three *ntaitr12356* quintuple mutants, and the seed germination rate of the wild type-I was also largely indistinguishable from that of the wild type (Figure 8A).

Clearly increased ABA sensitivity for all three *ntaitr12356* quintuple mutants was also observed in the seedling greening assays (Figure 8B). Quantitative assays show that in the presence of ABA, less than 20% of the seedlings of all the three *ntaitr12356* quintuple mutants produced green cotyledons, compared with ~60% for wild type and wild type-I seedlings (Figure 8C). Again, no difference was observed among the three *ntaitr12356* quintuple mutants, or between the wild type and the wild type-I (Figure 8C).

It has been shown that both the Arabidopsis AITRs and GmAITRs regulate ABA response via regulating the expression of some ABA signaling key regulator genes [12,13,33]. We thus examined if the expression levels of the ABA signaling key regulator genes may be changed in the *ntaitr12356* quintuple mutants. As the three *ntaitr12356* quintuple mutants showed a similar response to ABA in both the seed germination and seedling greening assays, we used only the *ntaitr12356-c3* mutant for the experiment. As shown in Figure 8D, the expression level of *NtPYL7*, an ABA receptor gene [43], was increased, whereas that of *NtPP2CA*, a PP2C phosphatase gene, was decreased in the *ntaitr12356-c3* mutant compared to the wild type seedlings. 

## 3. Discussion

ABA signaling via several key regulators activates/represses ABA responses genes, thereby regulating plant abiotic stress tolerance [6,7,8,9,10,11,12,13]. By characterizing unknown-function ABA response genes, we previously identified AITRs as a novel family of transcription repressors that negatively regulate abiotic stress tolerance in Arabidopsis [12,13]. As AITRs are conserved in angiosperms [12], we then characterized AITRs in several different crops to see if *AITRs* may be targeted for CRISPR/Cas9 gene-editing-based molecular breeding to improve abiotic stress tolerance in crops. Thus far, we found that *gmaitr* mutants generated by CRISPR/Cas9 gene editing of soybean *AITRs* showed moderately enhanced salt tolerance [33], but ABA sensitivity was slightly enhanced in the *gmaitr* mutants [33], opposite to that of the Arabidopsis *aitr* mutants [12], indicating that AITRs in different plants may have different functions, at least in some aspects.

We provide evidence in this study that NtAITRs shared similar features with AITRs characterized so far in other plants including Arabidopsis, tomato, cotton and soybean [12,32,33], i.e., *NtAITRs* are ABA response genes, as their expression levels were increased in response to ABA treatment (Figure 3), and NtAITRs function as transcription repressors, as the NtAITRs examined repressed the expression of the reporter gene in transfected protoplasts (Figure 4). It should be noted that due to the low expression level of *NtAITR2* and high nucleotide identity of the *NtAITRs* gene pairs (Figure S3), we failed to generated constructs for *NtAITR2* and *NtAITR5*, therefore transcription repression activities of NtAITR2 and NtAITR5 were not examined in this study. However, considering that NtAITRs shared high amino acid identity and similarity, and all have a partially conserved LxLxL motif (Figure 1), it is reasonable to assume that NtAITR2 and NtAITR5 can also function as transcription repressors, yet experimental evidence are required to examine if this is indeed the case. 

Our evidence in this study also show that even though NtAITRs shared similar features with AITRs in other plants, a difference in regulating ABA sensitivity was observed, as examined with the *ntaitr12356* quintuple mutants generated by CRISPR/Cas9 gene editing (Figure 6). Greatly increased ABA sensitivity was observed in the *ntaitr12356* quintuple mutants as compared with the wild type tobacco plants (Figure 8), opposite to that observed in the Arabidopsis *aitr* mutants, which showed greatly decreased ABA sensitivity [12,13]. On the other hand, NtAITRs may have similar functions as GmAITRs in regulating ABA sensitivity, as slightly enhanced ABA sensitivity was observed in the *gmaitr* mutants [33]. There are six genes in Arabidopsis but eight in cotton encoding AITRs [12,33], and there are also eight in tobacco (Figure 1). Considering that the maximum decrease in ABA sensitivity in Arabidopsis *aitr* mutants was observed in *aitr256* triple mutants [13], and the *ntaitr* mutants used this study are *ntaitr12356* quintuple mutants (Figure 6), whereas increased ABA sensitivity observed the *gmaitr* mutants may be only caused by the loss of function of two *GmAITR* genes [33], it is very likely that high-order mutants of *GmAITRs* may also result in greatly increased ABA sensitivity in soybean, i.e., NtAITRs and GmAITRs may have similar functions in regulating ABA sensitivity. 

Our results show that so far, all the AITRs examined in different plants negatively regulate abiotic stress tolerance, Arabidopsis *aitr236* triple and high-order mutants showed greatly enhanced tolerance to drought and salt [12,13], *gmaitr* mutants showed moderately enhanced tolerance to salt [33], and *ntaitr12356* quintuple mutants showed greatly enhanced tolerance to drought (Figure 7). These results provided evidence that *ATIRs* in crops indeed could be good targets for CRISPR/Cas9 gene editing to improve abiotic stress tolerance. 

As an industrial crop with great economic importance, tobacco’s yield and quality are affected by abiotic stresses [36,37,39,40,41]. Breeding of tobacco plants with enhanced abiotic stress tolerance will increase their yield and quality. As ABA affects plant responses to abiotic stresses via regulating ABA response genes [6,7,8,9,10,11,12,13], characterization of unknown-function ABA response genes may enable the identification of novel regulators of abiotic stress tolerance. Our evidence in this study indicates that NtAITRs are this kind of regulators. As the *ntaitr12356* quintuple mutants showed enhanced sensitivity to ABA (Figure 8), it is very likely that the functions of NtAITRs in regulating abiotic stress tolerance in tobacco are due to its feedback regulating roles of ABA responses. Yet, detailed mechanisms of NtAITRs in regulating abiotic stress tolerance in tobacco need to be further investigated.

Most importantly, our evidence in this study clearly indicates that it is not only possible but also practical to used *NtAITRs* as targets for CRISPR/Cas9 editing to improve abiotic stress tolerance in tobacco. First, coding sequences of *NtAITRs* shared high nucleotide identity (Figure S3), therefore it will be easier to identify target sequences that can target at least a pair of *NtAITR* genes. As an example, we identified four target sequences that can be used to target five *NtAITR* genes (Figure 5). Second, it is not difficult to generate transgene-free gene-edited mutants for *NtAITRs*, which may avoid the difficulty of using transgenic plants in production. Even though not all the target sites were edited, we obtained mutants with five *NtAITR* genes being edited (Figure 6), and we successfully isolated transgene-free mutants by using flowering phenotypes caused by the *FT* expression cassette in the *CRISPR/Cas9* construct (Figure 5). Third, greatly enhanced drought tolerance was observed in mutants with only some of the *NtAITR* genes being edited. The eight NtAITR proteins shared high amino acid sequence identity as well as conserved domains (Figure 1, Figure S2), suggesting that they may have redundant functions. However, greatly enhanced drought tolerance was already observed in the *ntaitr12356* quintuple mutants (Figure 7), indicating that it may be not necessary to edit all the *NtAITRs* to obtain tobacco plants with enhance abiotic stress tolerance. Yet, it will be of great interest to examine if further enhanced abiotic tolerance can be achieved if all the *NtAITRs* are being edited and if the loss of function of all *NtAITRs* may have any fitness cost.

In summary, our results in this study show that the expression of *NtAITRs* is induced by ABA and that NtAITRs function as transcription repressors and they positively regulate ABA sensitivity but negatively regulate drought tolerance in tobacco. As a result, *NtAITRs* can be used as targets for CRISPR/Cas9 gene-editing-based molecular breeding to improve abiotic stress tolerance in tobacco.

## 4. Materials and Methods

### 4.1. Identification of AITRs in Tobacco

To identify NtAITRs in tobacco, the entire amino acid sequences of Arabidopsis AITRs were used for Protein BLASTing on NCBI (https://blast.ncbi.nlm.nih.gov/Blast.cgi, accessed on 30 October 2018) with *N. tabacum* as selected organism. The full-length amino acid sequences of the retrieved NtAITRs were then used in reiterative BLASTing until no more NtAITRs were identifiable. The coding sequences of the *NtAITRs* were obtained by using the locus numbers to conduct gene searching on the KEGG GENES Database (https://www.genome.jp/kegg/genes.html, accessed on 30 October 2018), and corresponding genome sequences were obtained by BLASTing the *N. tabacum* genome with the coding sequences on the Sol Genomics Network (https://solgenomics.net/organism/Nicotiana_tabacum/genome, accessed on 30 October 2018). Full-length amino acid sequences of obtained NtAITRs were subjected to phylogenetic analysis (http://hylogeny.fr/phylo_cgi/index.cgi, accessed on 1 September 2022) with AITRs from Arabidopsis and soybean [12,33], and motif analysis was performed using MEME with default settings [44].

### 4.2. Plant Materials and Growth Conditions

The K326 tobacco was used as wild type for plant transformation and as control for ABA and drought treatment and drought tolerance assays. The *ntaitr12356* quintuple mutants were generated by CRISPR/Cas9 gene editing. The non-edited plant wild type-I was segregated from the offspring of the T0 plant from which the *ntaitr12356-c3* quintuple mutant was isolated and was also used as a control for ABA and drought treatment. The Col wild type Arabidopsis was used for protoplast isolation. The tobacco seedlings used for RNA isolation and ABA treatments were grown on ½ MS plates, and tobacco plants used for plant transformation, drought treatment and drought tolerance assays were grown in soil pots at 22–24 °C in a growth room with a 16/8 h (light/dark) photoperiod, as described previously [45]. 

### 4.3. ABA and Drought Treatment, RNA Isolation and RT-PCR

To examine the expression of *NtAITRs* and ABA signaling component genes in response to ABA, 10-day-old tobacco seedlings were treated with 50 μM ABA for 4 h in darkness on a shaker at 40 rpm and frozen in liquid N_2_ immediately for RNA isolation. To examine the expression of *NtAITRs* in response to drought treatment, 20-day-old soil-grown K326 wild type tobacco seedlings were watered with 20% PFG6000 and seedlings were collected 6 h after and frozen in liquid N_2_ immediately for RNA isolation.

Total RNA was isolated by using a Plant RNA kit (OMEGA BIO-TEK, Norcross, GA, USA) following the manufacturer’s instructions, and 1–2 μg total RNA was subjected to first-strand cDNA synthesis by using an EasyScript First-strand DNA Synthesis Super Mix (TransGen Biotech, Beijing, China). RT-PCR analysis was performed by using the synthesized cDNA as templates. The expression of the *NtGAPDH* gene was used as an internal reference control [46]. The primers used were listed in Table S1.

### 4.4. Constructs

The reporter construct of *LexA-Gal4:GUS* and the effector constructs *GD* and *LD-VP* were as described previously [47,48]. 

To generate *35S:GD-NtAITRs* constructs for protoplast transfection assays, the full-length open reading frame (ORF) of the *NtAITRs* was amplified by RT-PCR using RNA isolated from tobacco seedlings and cloned in frame with an N-terminal GD tag into the *pUC19* vector with the *35S* promoter [47,48]. 

In order to generate CRISPR/Cas9 constructs for *NtAITRs* gene editing, a total of four different target sequences within the exons of *NtAITRs* that may be simultaneously target five genes were selected on CRISPRscan (http://www.crisprscan.org/?page=sequence, accessed on 30 October 2018), and target specificity was evaluated on cas-offinder (http://www.rgenome.net/cas-offinder, accessed on 30 October 2018). The four target sequences are the following: 5′-GCACCTAAACCGGTCGCCGG-3′ for *NtAITR1* and *NtAITR3*, 5′-GCTCCTAAACCAGCCGCTGG-3′ for *NtAITR5* and *NtAITR6*, 5′-GCCGTGTGATGTGTGGAAGA-3′ for *NtAITR1*, *NtAITR2* and *NtAITR3*, and 5′-GCCGTGTGATGTGTGGAGAA-3′ for *NtAITR5* and *NtAITR6*. The four targets were inserted in to the *pHEE* vector with an *FT* expression cassette as reported previously [42]. The primers used were listed in Table S1. 

### 4.5. Plasmid Isolation, Protoplast Isolation and Transient Transfection

Plasmid isolation, protoplast isolation and transfection, and GUS activity assays were performed by following the procedure described previously [12,48,49,50]. Briefly, plasmid DNA of the reporter and effector constructs was isolated by using an EndoFree Plasmid Maxi Kit (OMEGA BIO-TEK). Protoplasts were isolated from leaves collected from ~3-week-old Col wild-type Arabidopsis and co-transfected with the *LexA-Gal4:GUS* reporter, the *LD-VP* and the *GD-NtAITRs* effector plasmid DNA. The transfected protoplasts were incubated for 20–22 h under darkness at room temperature. GUS activities were measured by using a SynergyTM HT fluorescence microplate reader (BioTEK, Winooski, VT, USA).

### 4.6. Generation of Transgene-Free ntaitr Mutants

Five- to six-week-old K326 tobacco plants were used for Agrobacterium *GV3101*-mediated stable transformation by infiltration, as described previously [45]. T0 transgenic seedlings generated were transferred into soil pots and grown in a growth room. The plants with early flowering phenotypes were selected, and gene editing status was detected by PCR amplification and sequencing of the DNA sequence fragments of *NtAITR1*, *NtAITR2*, *NtAITR3*, *NtAITR5* and *NtAITR6*. Transgene-free homozygous mutant plants were isolated in T1 or T2 progeny based on flowering phenotype and sequencing, as previously described [42,51].

### 4.7. Seed Germination and Seedling Greening Assays

Seed germination and cotyledon greening assays were used to examine ABA sensitivity of the *ntaitr12356* mutants obtained, and the K326 wild type and the wild type-I tobacco were used as controls. Healthy and uniform-sized seeds of the K326 wild type, the wild type-I and the *ntaitr12356* mutants were surface sterilized and germinated on 1/2 MS plates in the presence or absence of 2 μM ABA. Germinated seeds were counted daily after the transfer. Pictures were taken 14 days after the transfer, and seedlings with green cotyledons were counted. 

### 4.8. Drought Tolerance Assays

Drought tolerance assays of soil-grown tobacco were performed by using the procedures described previously [52,53,54] with some modification. Seeds of the K326 wild type, the wild type I and the *ntaitr12356* mutants were sown in soil trays and grown for 10 days with sufficient watering. The plants were then subjected to drought treatment by withholding watering for 20 days, and watering was resumed. Pictures were taken before drought treatment, after drought treatment and 5 days after watering was resumed.

## Data Availability

All data obtained were presented in this article and the supporting information.

## Supplementary Materials

The following are available online at https://www.mdpi.com/article/10.3390/ijms232315268/s1.

## References

[1] Wang W., Vinocur B., Altman A. (2003). Plant responses to drought, salinity and extreme temperatures: Towards genetic engineering for stress tolerance. Planta.

[2] Fujita M., Fujita Y., Noutoshi Y., Takahashi F., Narusaka Y., Yamaguchi-Shinozaki K., Shinozaki K. (2006). Crosstalk between abiotic and biotic stress responses: A current view from the points of convergence in the stress signaling networks. Curr. Opin. Plant Biol..

[3] Boyer J.S. (1982). Plant productivity and environment. Science.

[4] Ghosh D., Xu J. (2014). Abiotic stress responses in plant roots: A proteomics perspective. Front. Plant Sci..

[5] Yamaguchi-Shinozaki K., Shinozaki K. (2006). Transcriptional regulatory networks in cellular responses and tolerance to dehydration and cold stresses. Annu. Rev. Plant Biol..

[6] Rodriguez P.L., Leube M.P., Grill E. (1998). Molecular cloning in Arabidopsis thaliana of a new protein phosphatase 2C (PP2C) with homology to ABI1 and ABI2. Plant Mol. Biol..

[7] Gosti F., Beaudoin N., Serizet C., Webb A.A., Vartanian N., Giraudat J. (1999). ABI1 protein phosphatase 2C is a negative regulator of abscisic acid signaling. Plant Cell.

[8] Fujii H., Verslues P.E., Zhu J.K. (2007). Identification of two protein kinases required for abscisic acid regulation of seed germination, root growth, and gene expression in Arabidopsis. Plant Cell.

[9] Umezawa T., Nakashima K., Miyakawa T., Kuromori T., Tanokura M., Shinozaki K., Yamaguchi-Shinozaki K. (2010). Molecular basis of the core regulatory network in ABA responses: Sensing, signaling and transport. Plant Cell Physiol..

[10] Guo J., Yang X., Weston D.J., Chen J.G. (2011). Abscisic acid receptors: Past, present and future. J. Integr. Plant Biol..

[11] Dong T., Park Y., Hwang I. (2015). Abscisic acid: Biosynthesis, inactivation, homoeostasis and signalling. Essays Biochem..

[12] Tian H., Chen S., Yang W., Wang T., Zheng K., Wang Y., Cheng Y., Zhang N., Liu S., Li D. (2017). A novel family of transcription factors conserved in angiosperms is required for ABA signalling. Plant Cell Environ..

[13] Chen S., Zhang N., Zhou G., Hussain S., Ahmed S., Tian H., Wang S. (2021). Knockout of the entire family of AITR genes in Arabidopsis leads to enhanced drought and salinity tolerance without fitness costs. BMC Plant Boil..

[14] Fujita Y., Nakashima K., Yoshida T., Katagiri T., Kidokoro S., Kanamori N., Umezawa T., Fujita M., Maruyama K., Ishiyama K. (2009). Three SnRK2 protein kinases are the main positive regulators of abscisic acid signaling in response to water stress in Arabidopsis. Plant Cell Physiol..

[15] Park S.Y., Peterson F.C., Mosquna A., Yao J., Volkman B.F., Cutler S.R. (2015). Agrochemical control of plant water use using engineered abscisic acid receptors. Nature.

[16] Yoshida T., Fujita Y., Maruyama K., Mogami J., Todaka D., Shinozaki K., Yamaguchi-Shinozaki K. (2015). Four Arabidopsis AREB/ABF transcription factors function predominantly in gene expression downstream of SnRK2 kinases in abscisic acid signaling in response to osmotic stress. Plant Cell Environ..

[17] Zhao Y., Chan Z., Gao J., Xing L., Cao M., Yu C., Hu Y., You J., Shi H., Zhu Y. (2016). ABA receptor PYL9 promotes drought resistance and leaf senescence. Proc. Natl. Acad. Sci. USA.

[18] Bueso E., Rodriguez L., Lorenzo-Orts L., Gonzalez-Guzman M., Sayas E., Muñoz-Bertomeu J., Ibañez C., Serrano R., Rodriguez P.L. (2014). The single-subunit RING-type E3 ubiquitin ligase RSL1 targets PYL4 and PYR1 ABA receptors in plasma membrane to modulate abscisic acid signaling. Plant J..

[19] Seo K.I., Lee J.H., Nezames C.D., Zhong S., Song E., Byun M.O., Deng X.W. (2014). ABD1 is an Arabidopsis DCAF substrate receptor for CUL4-DDB1-based E3 ligases that acts as a negative regulator of abscisic acid signaling. Plant Cell.

[20] Yu F., Lou L., Tian M., Li Q., Ding Y., Cao X., Wu Y., Belda-Palazon B., Rodriguez P.L., Yang S. (2016). ESCRT-I component VPS23A affects ABA signaling by recognizing ABA receptors for endosomal degradation. Mol. Plant..

[21] Belda-Palazon B., Rodriguez L., Fernandez M.A., Castillo M.C., Anderson E.A., Gao C., Gonzalez-Guzman M., Peirats-Llobet M., Zhao Q., De Winne N. (2016). FYVE1/FREE1 interacts with the PYL4 ABA receptor and mediates its delivery to the vacuolar degradation pathway. Plant Cell.

[22] Stone S.L., Williams L.A., Farmer L.M., Vierstra R.D., Callis J. (2006). KEEP ON GOING, a RING E3 ligase essential for Arabidopsis growth and development, is involved in abscisic acid signaling. Plant Cell.

[23] Chen Y.T., Liu H.X., Stone S., Callis J. (2013). ABA and the ubiquitin E3 ligase KEEP ON GOING affect proteolysis of the Arabidopsis thaliana transcription factors ABF1 and ABF3. Plant J..

[24] Liu H., Stone S.L. (2010). Abscisic acid increases Arabidopsis ABI5 transcription factor levels by promoting KEG E3 ligase self-ubiquitination and proteasomal degradation. Plant Cell.

[25] Liu H., Stone S.L. (2013). Cytoplasmic degradation of the Arabidopsis transcription factor abscisic acid insensitive 5 is mediated by the RING-type E3 ligase KEEP ON GOING. J. Biol. Chem..

[26] Kong L., Cheng J., Zhu Y., Ding Y., Meng J., Chen Z., Xie Q., Guo Y., Li J., Yang S. (2015). Degradation of the ABA co-receptor ABI1 by PUB12/13 U-box E3 ligases. Nat. Commun..

[27] Jung C., Seo J.S., Han S.W., Koo Y.J., Kim C.H., Song S.I., Nahm B.H., Choi Y.D., Cheong J.J. (2008). Overexpression of AtMYB44 enhances stomatal closure to confer abiotic stress tolerance in transgenic Arabidopsis. Plant Physiol..

[28] Liu S., Hu Q., Luo S., Li Q., Yang X., Wang X., Wang S. (2015). Expression of wild-type PtrIAA14.1, a poplar Aux/IAA gene causes morphological changes in Arabidopsis. Front. Plant Sci..

[29] Huang Y., Feng C.Z., Ye Q., Wu W.H., Chen Y.F. (2016). Arabidopsis WRKY6 transcription factor acts as a positive regulator of abscisic acid signaling during seed germination and early seedling development. PLoS Genetics.

[30] Wang X., Wang W., Wang Y., Zhou G., Liu S., Li D., Adnan, Hussain S., Ahmed S., Zhang C. (2020). AIW1 and AIW2, two ABA-induced WD40 repeat-containing transcription repressors function redundantly to regulate ABA and salt responses in Arabidopsis. J. Plant Interact..

[31] Cheng Y., Ma Y., Zhang N., Lin R., Yuan Y., Tian H., Hussain S., Chen S., Yang W., Li Y. (2022). The R2R3 MYB transcription factor MYB71 regulates abscisic acid response in Arabidopsis. Plants.

[32] Wang T., Dong Q., Wang W., Chen S., Cheng Y., Tian H., Li X., Hussain S., Wang L., Gong L. (2021). Evolution of AITR family genes in cotton and their functions in abiotic stress tolerance. Plant Biol..

[33] Wang T., Xun H., Wang W., Ding X., Tian H., Hussain S., Dong Q., Li Y., Cheng Y., Wang C. (2021). Mutation of *GmAITR* genes by CRISPR/Cas9 genome editing results in enhanced salinity stress tolerance in soybean. Front. Plant Sci..

[34] Andrianov V., Borisjuk N., Pogrebnyak N., Brinker A., Dixon J., Spitsin S., Flynn J., Matyszczuk P., Andryszak K., Laurelli M. (2010). Tobacco as a production platform for biofuel: Overexpression of Arabidopsis DGAT and LEC2 genes increases accumulation and shifts the composition of lipids in green biomass. Plant Biotechnol. J..

[35] Barla F.G., Kumar S. (2016). Tobacco biomass as a source of advanced biofuels. Biofuels.

[36] de las Mercedes Dana M., Pintor-Toro J.A., Cubero B. (2006). Transgenic tobacco plants overexpressing chitinases of fungal origin show enhanced resistance to biotic and abiotic stress agents. Plant Physiol..

[37] Wei W., Zhang Y., Han L., Guan Z., Chai T. (2008). A novel WRKY transcriptional factor from *Thlaspi caerulescens* negatively regulates the osmotic stress tolerance of transgenic tobacco. Plant Cell Rep..

[38] Yang C., Wang R., Gou L., Si Y., Guan Q. (2017). Overexpression of *Populus trichocarpa* mitogen-activated protein kinase kinase4 enhances salt tolerance in tobacco. Int. J. Mol. Sci..

[39] Gao Y., Yang J., Duan W., Ma X., Qu L., Xu Z., Yang Y., Xu J. (2022). NtRAV4 negatively regulates drought tolerance in *Nicotiana tabacum* by enhancing antioxidant capacity and defence system. Plant Cell Rep..

[40] Sun H., Sun X., Wang H., Ma X. (2020). Advances in salt tolerance molecular mechanism in tobacco plants. Hereditas.

[41] Xie H., Bai G., Lu P., Li H., Fei M., Xiao B., Chen X., Tong Z., Wang Z., Yang D. (2022). Exogenous citric acid enhances drought tolerance in tobacco (*Nicotiana tabacum*). Plant Biol..

[42] Cheng Y., Zhang N., Hussain S., Ahmed S., Wang S. (2019). Integration of a FT expression cassette into CRISPR/Cas9 construct enables fast generation and easy identification of transgene-free mutants in Arabidopsis. PLoS ONE.

[43] Bai G., Xie H., Yao H., Li F., Chen X., Zhang Y., Xiao B., Yang J., Li Y., Yang D.H. (2019). Genome-wide identification and characterization of ABA receptor PYL/RCAR gene family reveals evolution and roles in drought stress in Nicotiana tabacum. BMC Genomics.

[44] Bailey T.L., Boden M., Buske F.A., Frith M., Grant C.E., Clementi L., Ren J., Li W.W., Noble W.S. (2009). MEME SUITE: Tools for motif discovery and searching. Nucleic Acids Res..

[45] Sparkes I.A., Runions J., Kearns A., Hawes C. (2006). Rapid, transient expression of fluorescent fusion proteins in tobacco plants and generation of stably transformed plants. Nat. Protoc..

[46] Wang Z., Wang S., Wu M., Li Z., Liu P., Li F., Chen Q., Yang A., Yang J. (2019). Evolutionary and functional analyses of the 2-oxoglutarate-dependent dioxygenase genes involved in the flavonoid biosynthesis pathway in tobacco. Planta.

[47] Tiwari S.B., Hagen G., Guilfoyle T.J. (2004). Aux/IAA proteins contain a potent transcriptional repression domain. Plant Cell.

[48] Wang S., Tiwari S.B., Hagen G., Guilfoyle T.J. (2005). Auxin response factor 7 restores the expression of auxin-responsive genes in mutant Arabidopsis leaf mesophyll protoplasts. Plant Cell.

[49] Wang X., Wang X., Hu Q., Dai X., Tian H., Zheng K., Wang X., Mao T., Chen J.G., Wang S. (2015). Characterization of an activation-tagged mutant uncovers a role of GLABRA2 in anthocyanin biosynthesis in Arabidopsis. Plant J..

[50] Dai X., Zhou L., Zhang W., Cai L., Guo H., Tian H., Schiefelbein J., Wang S. (2016). A single amino acid substitution in the R3 domain of GLABRA1 leads to inhibition of trichome formation in Arabidopsis without affecting its interaction with GLABRA3. Plant Cell Environ..

[51] Liu Y., Zeng J., Yuan C., Guo Y., Yu H., Li Y., Huang C. (2019). Cas9-PF, an early flowering and visual selection marker system, enhances the frequency of editing event occurrence and expedites the isolation of genome-edited and transgene-free plants. Plant Biotechnol. J..

[52] Chen Y., Han Y., Zhang M., Zhou S., Kong X., Wang W., Min Y.Z. (2016). Overexpression of the wheat expansin gene *TaEXPA2* improved seed production and drought tolerance in transgenic tobacco plants. PLoS ONE.

[53] Xia Z., Xu Z., Wei Y., Wang M. (2018). Overexpression of the maize sulfite oxidase increases sulfate and GSH Levels and enhances drought tolerance in transgenic tobacco. Front. Plant Sci..

[54] Li X., Wang Q., Guo C., Sun J., Li Z., Wang Y., Yang A., Pu W., Guo Y., Gao J. (2022). NtNAC053, a novel NAC transcription factor, confers drought and salt tolerances in tobacco. Front. Plant Sci..

