# Supplementary material for: CRISPR/Cas9 Gene Editing of NtAITRs, a Family of Transcription Repressor Genes, Leads to Enhanced Drought Tolerance in Tobacco
Source: Int J Mol Sci. 2022 Dec 3;23(23):15268. doi: 10.3390/ijms232315268 (PMC9737578; doi:10.3390/ijms232315268)
Supplement: Supplementary file 1 [file ijms-23-15268-s001.zip › ijms-2005789-supplementary.pdf]

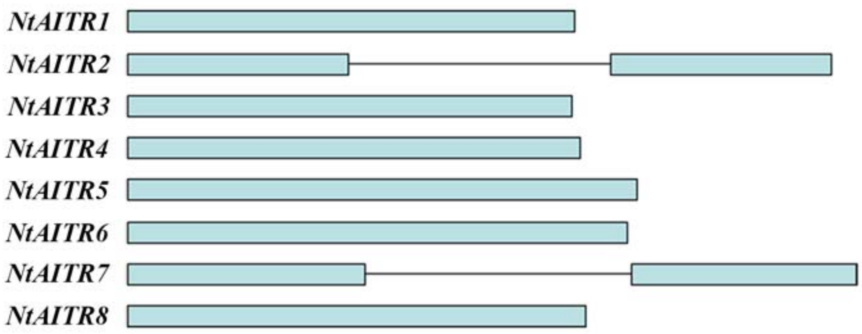

Figure S1. Diagram of gene structure of *NtAITRs*.

Boxes indicate exons, and lines indicate introns.

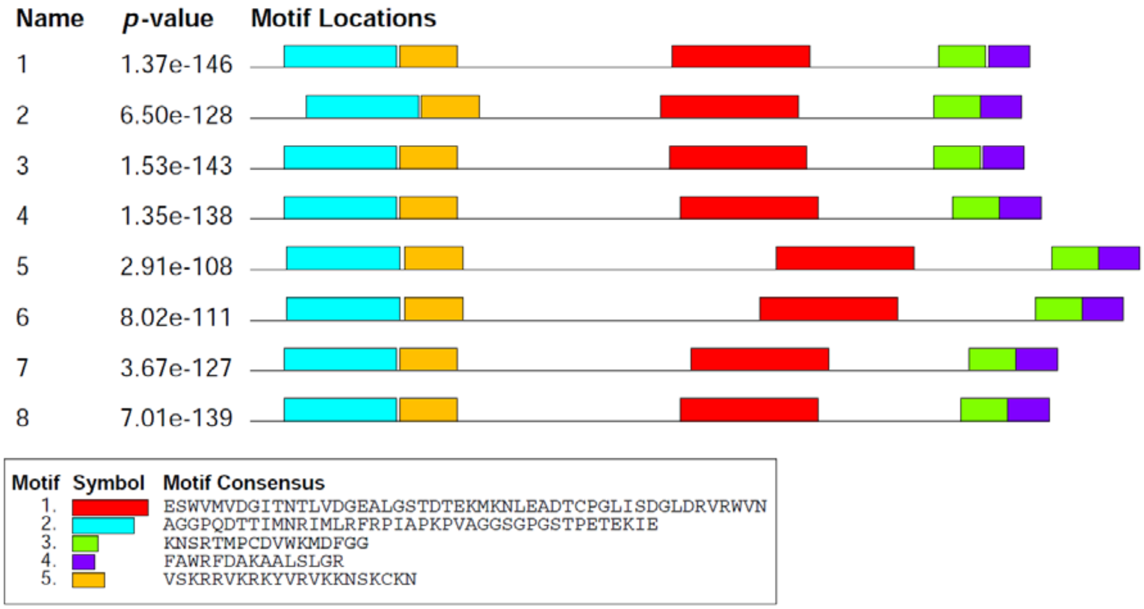

Figure S2. Conserved domains in NtAITRs.

Motif analysis was performed using MEME.

```
NtAITR1  ------------------------ATGGACGGTGGAAATGGTTGGCCCGC  26
NtAITR3  ------------------------ATGGACGGTGGAAATGGTTGGCCAGC  26
NtAITR2  ATGAGACAGAGCTCTTCAACAGGTATGGATGTTGGGGATGGTGGGCCCTC  50
NtAITR7  ------------------------ATGGATGTTGAGAATAGTGGGCCCTC  26
NtAITR4  ------------------------ATGGACATCGAAAACAATTGGGCCGG  26
NtAITR8  ------------------------ATGGACGTCGGAAACAATTGGGCCGG  26
NtAITR5  ------------------------ATGGATGGTAGAGGAGGGTGTTGTAT  26
NtAITR6  ------------------------ATGGATGGTAGAGGAGGGTGTTGTAT  26

NtAITR1  CGCTAGATACGCCGG---TGGACCGCAGGATACGACGATAAAGAACCGGA  73
NtAITR3  CGCTAGATACGCCGG---TGGACCGCAGGATACAATGATAATGAACAATA  73
NtAITR2  CGATGGATGCCACGT---TGGACCTTATGATACGACGATAATGAACCGGA  97
NtAITR7  CTATGGATGCCACGT---TGGACCACAAGATAGGATGATAATGAACCGGA  73
NtAITR4  CCCAGAATACTCAGG---CGCACCAAAAGATATGACAATAATCAACAAGA  73
NtAITR8  GTCTGGCTACCCCGG---TGCACCTAAAGATATGACGATAATCAACAAGA  73
NtAITR5  TGCTAGGTACGCAGGGGGTGGTGCGTACGATATGTCAAAAGTGGACCGGA  76
NtAITR6  TGCTAGGTACGCAGGGGGTGGTGCGTACGATATGTCAAAAGTGGACCGGA  76

NtAITR1  TAATGCTAAGATTCCGACCGATCGCACCTAAACCGGTCGCCGGGGGTTCA  123
NtAITR3  TAATGCTAAGATTCCGACCGATCGCACCTAAACCGGTCGCCGGGGGTTCA  123
NtAITR2  TAATGTTAAGATTCCGGCCGATTGCGCCGAAACCTGTTGCCGACGGTTCA  147
NtAITR7  TAATGTTAAGATTCCGGCCGATTGCACCGAAACCGGTCGCCGACGGTTCA  123
NtAITR4  TGATGCTAAGATTCCGGCCGATTGCGCCGAAACCCGTCGCTAATTCTTCA  123
NtAITR8  TGATGCTAAGATTCCGGCCGATTGCGCCGAAGCCCGTCGCTAATTCTTCG  123
NtAITR5  TTATGCTCAGATTCAGACCCATCGCTCCTAAACCAGCCGCTGGTGGGTCT  126
NtAITR6  TAATGCTCAGATTCAGACCCATCGCTCCTAAACCAGCCGCTGGTGGGTCT  126

NtAITR1  TCTCCTGGTTCAACACCGGAAGGAAACAAGATAGAA---CTTGTTACCAA  170
NtAITR3  TCTCCTGGTTCAACACCGGAAGGAAACAAGATAGAA---CTTGTTACCAA  170
NtAITR2  GGTCCTGGTTCTAAACCGAAGAGTGAAAAGGCTGAA---CCGGTCAGCAA  194
NtAITR7  GGTCCGGGTTCTAAACCGGAGAATGAAAAGGCAGAA---CCGGTTAGCAG  170
NtAITR4  GGCTCTGGCTCTACGATCGAGACTCACGAGATTGAA---GTGGTTAGCAA  170
NtAITR8  GGCTCTGGCTCTACGATCGAGACTCATAAGATTGAA---GTGGTTAGCAA  170
NtAITR5  GTTTCCGGTGCTTCTACTCCGCCACAAAAGACTGAGGTTCCTGTTCGTAC  176
NtAITR6  GTTTCCGGCGCTTCTACTCCTCCACAAAAGACTGAGGCTCCTGTTCGTAC  176

NtAITR1  ACGAAGAGTCAAAAGAAAGTACGTTAGAGTTAAGAAAAA------TAGCA  214
NtAITR3  ACGAAGAGTCAAAAGAAAGTACGTTAGAGTTAAGAAAAA------TAGCA  214
NtAITR2  ACGGAGAGGGAAGAGAAAGTACGTTAGAGTTAAGAAAAG------TAGGA  238
NtAITR7  ACGAAGAGGGAAGAGAAAGTACGTTAGAGTTAAGAAAAA------TAGGA  214
NtAITR4  AAGAAGAACAAAGAGAAAGTACGTTAGAGTTAAGAAAAA------TAGCA  214
NtAITR8  AAGAAGAACAAAGAGAAAGTACGTTAGAGTTAAGAAAAA------TAGCA  214
NtAITR5  GGGTCGGTGGAAACGAAGGTACGTCAAAGATAATAAAAGTAGTACTAGTA  226
NtAITR6  GGGCCGTTGGAAGCGAAGGTACGTTAAAGATAATAAAAACACTAGTAGCA  226

NtAITR1  AGTT---CAAAAG---TAACAAAGAAG-------------------AAGA  239
NtAITR3  AGTG---CAAAAG---TAACAAAGAAG-------------------AAGG  239
NtAITR2  AGTGTACTAACAA---TAGTACAGAGA-------------------AAGA  266
NtAITR7  AGTG---TAAGAA---TAGTGCAGAGA-------------------AAGA  239
NtAITR4  ACTA---CAAGAA---TAAAAAAGAAG-------------------ACAA  239
NtAITR8  ATTA---CAAGAA---TAAAAAAGAAA-------------------ACAG  239
NtAITR5  GCTCTAACAAGAGGTCTAGTAGTGGAAGTCGTAGTCCTGCTGGTCGAAAA  276
NtAITR6  CTTCTAACAAGAGATCTAGTAGCGGAAGTCGTAGTCCTAGTGGTAGAAAA  276

NtAITR1  AGGAGAAAA--------------GGACGGATCCTTACAG---TTTACTGA  272
NtAITR3  AGAAAAGGA-----------------CGGATCCTTACAG---TTTACTGA  269
NtAITR2  AGAAAGAAAATT-----------AGATGGATCTTTGAAGAAGTTTAATGA  305
NtAITR7  AGAAAGAAA--------------AGATGGATCT------------GATAA  263
NtAITR4  AGAAACAAA--------------AGATGGATCCTTAGTT---TTGGATGA  272
NtAITR8  AGAAAAAAA--------------AGAAGGATCTTTAGTT---TTGGATGA  272
NtAITR5  AGGAAGGCACCTTCGCCTGAGGAAAATGAGTCCAACGGTAAGACTGTATC  326
NtAITR6  AGGAAGGCATCTTCACCTGAGGAAAATGAGTCCAACGGTAAGACTGTATC  326
```

```
NtAITR1  TAGTCAA-------------A-----CTGTTTTAACCCTTCAGTTGATGC  304
NtAITR3  TAGTAAA-------------A-----CTGTTTTAACCCTTCAGTTGATGC  301
NtAITR2  TAATGGA-------------A-----GTGTAGTAACTCTTCAGCTACTAC  337
NtAITR7  TAATGGA-------------T-----CTATTGTAACCCTTCAGTTACTTC  295
NtAITR4  TCATGGA-------------A-----CAGTTGTAACACTACAACTACTGC  304
NtAITR8  TCATGGA-------------A-----CTATTGTAACACTTCAGCTACTGC  304
NtAITR5  TGGTGGTACAGTTTCTGGGGGCCAAGCGGTAGTTACCTTACCCTTATTAT  376
NtAITR6  TGGTGGG---------------CAAGCTGTAGTTACCTTACCCTTATTAT  361

NtAITR1  CTGAAAGC----AGTAGCAGCGTTA-AGAACTCTTTAGAAAAT--ATCGG  347
NtAITR3  CTGAAAGC----AGCAGCAGCGTTA-AGAACTCTCTAGAAAAT--ATCGG  344
NtAITR2  CGGAAAGT----AGCAGCAGCACTA-CTAACCTTCCAGATAATGGATCTT  382
NtAITR7  CGGAGAGT----AGCAGCAGTGTTA-CAAACCCGACAGATAACGTTTCTT  340
NtAITR4  CAGAAAGT----AGTGGAGGAGTTA-AACGCTCACCAGATAACAGATCTT  349
NtAITR8  CAGAAACT----AGTGGAGGAGTTA-AAAACTCACCAGAGAACAGATCTT  349
NtAITR5  CCGAGTCTCCTGAAAGGAAGGGTTCTCCTGTCGATCATTCAGTAGGTTTT  426
NtAITR6  CAGAGTCTCCTGAACGGAAGGACTCTCCTGGCGATCATTCAGTAGGTTTG  411

NtAITR1  ATCTAGA-------CCTTTTT----GGATGAATT----------------  370
NtAITR3  ATCTAGA-------CCTTTTT----GGATGAATT----------------  367
NtAITR2  TTCAAAAGGAAATCACTTTCTC-TGAGCTAGATC----------------  415
NtAITR7  TTCAAAAAGAAATCTCTTTCTC-TGAGCTAGATCAACCCCAATGGATGAG  389
NtAITR4  TCCCAAAAACCATCAATTTTT----GGATGAATT----------------  379
NtAITR8  ACCCAAAAACCATCAATTTTT----GGATGAATT----------------  379
NtAITR5  GTGATAAAACCAGAAAAATATGCTCCGATATGGTTAAACTTTGGTAGCCA  476
NtAITR6  GTGAAAAAACCAGAAAAATATACTCCGATCTGGTTAAACTTTGGTAGCCA  461

NtAITR1  --TTCAGAAACCGGAGAATAACGATGTTTTAACAGAAGGATCAGTGGATC  418
NtAITR3  --TTCAGAAATCGGAGAATAACGATGTTTTAGCCGTAGGATCAGTGGATC  415
NtAITR2  --------------------------------------------------  415
NtAITR7  TTTTCATGAATATGATAATAATGGTATATCCGCCTTCAGTTCAACGGATC  439
NtAITR4  --TTGATAAATCTGAGAATATCGATATTTTATCAATCGGTGCACCGAATC  427
NtAITR8  --TTGATAAATCTGAGAACAACGATATTTTATCAGTCGGTGCACCGAATC  427
NtAITR5  AGGGAATAATAATAATGATAATAGTCTGTTGCAGGGTTACGGAGGGGTTG  526
NtAITR6  AGGGAATAGTAAT---GATAATAGTCAGTTGCAGGGTTACGGAGGGGTTG  508

NtAITR1  GGATAGATCGGACGGTGGAGATA------CAGAAGAAAAGGGTTATAGAG  462
NtAITR3  AGATAGATCGGACGGTGGAGATG------CAGAAGAAAAGGGTTATAGAG  459
NtAITR2  ---------TGAAGGTGGCTATG------GAGACACCAAAAATGGTAGAA  450
NtAITR7  GGTTAGATCTGAAGGTGACAACG------CAGACACCAAGAATGGTAGAG  483
NtAITR4  TGTTAGATCGGACGGCGGAGATG------CGATCTGCGAAGGTAGTGGAG  471
NtAITR8  TGTTAGATCAGACGGTGGAGATG------CGATCTGCCAAGGTAGTGGAG  471
NtAITR5  GTATGGATCGTTCAGTAGTGATGTTGCCTCAGCCAGTAAGGGTAGTGGGG  576
NtAITR6  GTATGGATCGTTCAGTACTTATGTTGCCTCAGCCAGTAAGGGTAGTGGGG  558

NtAITR1  TCATGGGTGATGGTGGATAAAATGACAAACACGTTGGTAGATGGAGAAGC  512
NtAITR3  TCATGGGTGATGGTGGATAAAATGTCAAACACGTTGGTAGATGGAGAAGC  509
NtAITR2  TCTTGGGTGATGGTGGAAGGTATGACAAAAATGTTTGTAGATCGAGAAGC  500
NtAITR7  TCTTGGGTGATGGTGGATAGAATAACAAACATATTTGTGGATAGTGAAGG  533
NtAITR4  TCATGGGTGATGGTTGATGGTATAACAAACACTTTGGTAGATTTATCAGC  521
NtAITR8  TCATGGGTGATGGTTGATGGTATAACAAATACTTTGGTAAGTTTATCAGC  521
NtAITR5  TCATGGGTAAAGGTGGAAAGCGTGACTGACGCGTGGGCGGAAGGGTATGG  626
NtAITR6  TCATGGGTAAAGGTGGAAAGCGTGACTGACGCGTGGGTAGAAGGGTATGG  608

NtAITR1  GTTAGGGAGTACGGACATGGATAAGATGAAGAATCTGGAAGCTGACACGT  562
NtAITR3  GCTAGGGAGTACGGACATGGAGAAGATGAAGAATTTGGAAGCTGACACGT  559
NtAITR2  TTTAGGAAGTACAGATACAGAGAAGATGAAGAATCTAGAGAAAGACACCT  550
NtAITR7  CTTAGGAAGTACGGACATGGAGAAAATGAAGAATCTGGAGACGGACACGT  583
NtAITR4  CTTAGGAAGTACGGATACGGAGAAGATGAAGAATCTGGAAGCTGACACGT  571
NtAITR8  CTTAGGAAATACGGATACGGAGAAGATGAAGAATCTGGAAGCTGACACGT  571
NtAITR5  GCTAGGACGTACGGATGAGGACAAACTGGTGAATCTAGAGCGGGACAGCT  676
NtAITR6  GCTAGGACGTACAGATGAGGAGAAACTGGTAAATCTAGAGCTGGACAGCT  658
```

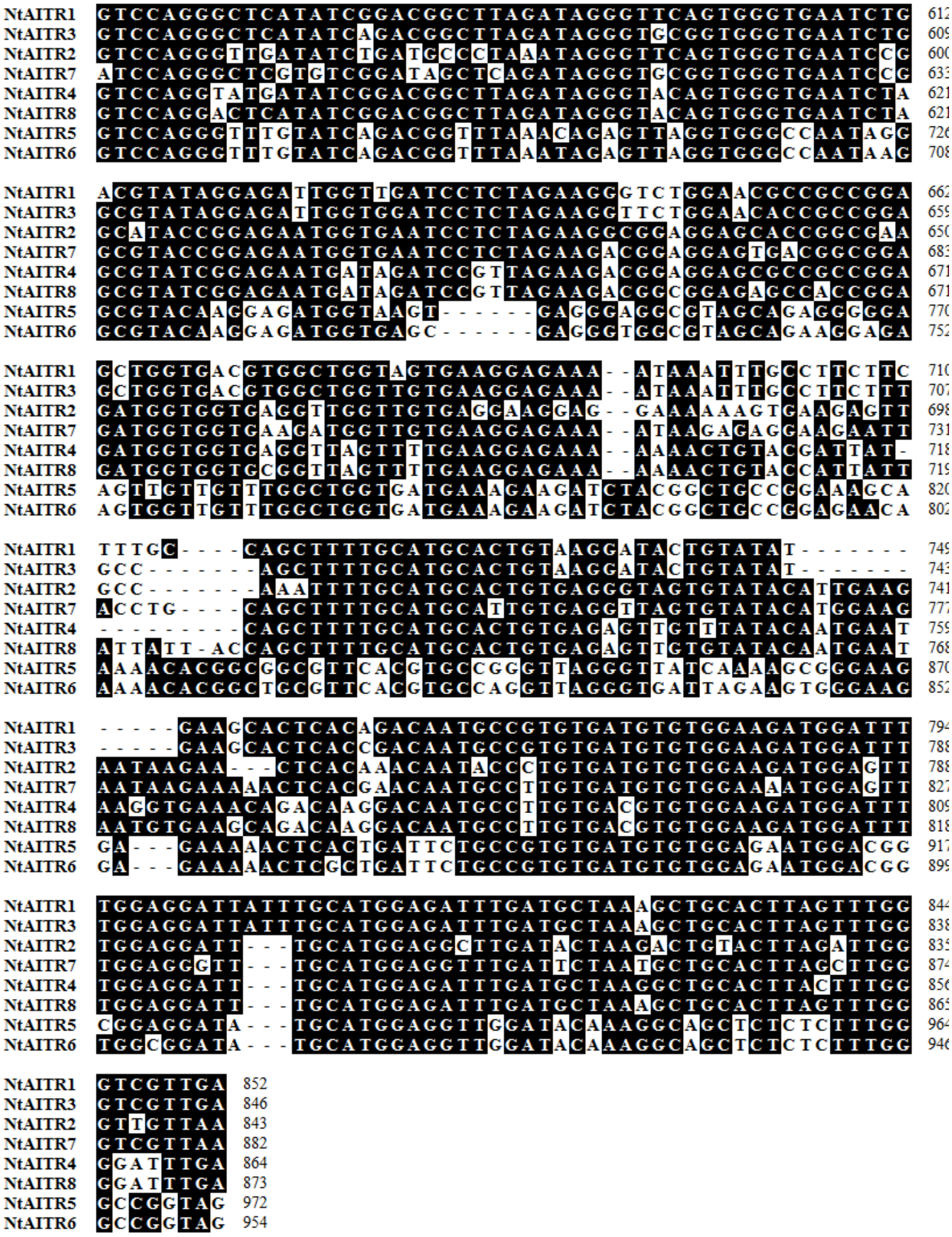

Figure S3. CDS sequences alignment of *NtAITRs*.

Sequence alignment was performed using BioEdit. Identical nucleotides were shaded in black.

Table S1. Primers used in this study

Primer	Sequence 5'-3'
NtAITR1/3-Nde1F	CAACATATGGACGGTGGAAATGGTTG
NtAITR1/3-Sac1R	CAAGAGCTCAACGACCCAAACTAAGTGC
NtAITR5/6-Nde1F	CAACATATGGATGGTAGAGGAGGGTG
NtAITR5/6-Sac1R	CAAGAGCTCCTACCGGCCCAAAGAGAGAG
NtAITR2-Nde1F	CAACATATGGATGTTGGGGATGGTGG
NtAITR2-Sac1R	CAAGAGCTCTTAACAACCCAATCTAAGTACAG
NtAITR4-Nde1F	CAACATATGGACATCGAAAACAATTGG
NtAITR4-Sac1R	CAAGAGCTCAAATCCCCAAAGTAAGTGC
NtAITR7-Nde1F	CAACATATGGATGTTGAGAATAGTGG
NtAITR7-Sac1R	CAAGAGCTCTTAACGACCCAAGCTAAGTGCAG
NtAITR8-Nde1F	CAACATATGGACGTCGGAAACAATTGG
NtAITR8-Sac1R	CAAGAGCTCAAATCCCCAAACTAAGTGC
NtAITR1-F	GTTTTAACAGAAGGATC
NtAITR2-F	TGAAGAAGTTTAATGATAATGGAAG
NtAITR3-F	CGATGTTTTAGCCGTAG
NtAITR5-R	TTATCATTATTATTATTCCCTTG
NtAITR6-R	CAACTGACTATTATCATTACTA
NtPYL7-F	ATGCTTCCCAATAGTCAGA
NtPYL7-R	CGGACTTCACGGAGAGTA
PP2CA-F	GCTGGTGATAATAATCTGTC
PP2CA-R	AGACGATAATCTTCTCGGA
NtGAPHD-F	TGGGTGTCAACGAGAAGGAA
NtGAPHD-R	TCTGGGTGGCAGTAAGGGA
Pps-GGL	TTCAGAggtctcTctcgACTAGTATGGAATCGGCAGCAAAGG
AtU6-1-sgRNA1-	CCGGCGACCGGTTTAGGTGCaatcactacttcgtct
gRT-sgRNA1+	CACCTAAACCGGTCGCCGGgttttagagctagaaat
Pgs-GG2	AGCGTGggtctcGtcagggTCCATCCACTCCAAGCTC
Pps-GG2	TTCAGAggtctcTctgacacTGGAATCGGCAGCAAAGG
AtU6-26-sgRNA2-	TCTTCCACACATCACACGGCaatcactacttcgactc
gRT-sgRNA2+	CCGTGTGATGTGTGGAAGAgttttagagctagaaat
Pgs-GG3	AGCGTGggtctcGtcttcacTCCATCCACTCCAAGCTC
Pps-GG3	TTCAGAggtctcTaagacttTGGAATCGGCAGCAAAGG
AtU6-29-sgRNA3-	CCAGCGGCTGGTTTAGGAGCaatctcttagtcgact
gRT-sgRNA3+	CTCCTAAACCAGCCGCTGGgttttagagctagaaat
Pgs-GG4	AGCGTGggtctcGagtccttTCCATCCACTCCAAGCTC
Pps-GG4	TTCAGAggtctcTgactacaTGGAATCGGCAGCAAAGG
AtU6-1-sgRNA4-	TTCTCCACACATCACACGGCaatcactacttcgtct
gRT-sgRNA4+	CCGTGTGATGTGTGGAGAAgttttagagctagaaat
Pgs-GGR	AGCGTGggtctcGaccgACGCGTATCCATCCACTCCAAGCTC
